# Improving the Combustion Efficiency of Aluminum-Based Composite, Al@IL/FG, Through Surface Activation Reaction

**DOI:** 10.3390/nano16120757

**Published:** 2026-06-16

**Authors:** Qi-Long Zheng, Zhi-Lei Huang, Hui-Xiang Xu, Ji-Zhen Li, Wei He

**Affiliations:** 1 Xi’an Modern Chemistry Research Institute, Xi’an 710065, China; xhx204@163.com (H.-X.X.); jizhenli@126.com (J.-Z.L.); 2 Key Laboratory of Advanced Spatial Mechanism and Intelligent Spacecraft, Ministry of Education, School of Aeronautics and Astronautics, Sichuan University, Chengdu 610065, China

**Keywords:** aluminum particle, solid propellant, combustion efficiency, fluorinated graphene, ionic liquid

## Abstract

Low combustion efficiency is a challenge of aluminum (Al) particles in solid propellants, especially in small solid rocket motors. Therefore, it is necessary to adjust the combustion performance of Al to improve the energy release of solid propellants. Here, a core–shell structured Al-based composite Al@IL/FG with high combustion efficiency has been prepared through ionic liquid (IL) and fluorinated graphene (FG) coating. It is seen that IL can form a smooth coating layer on the surface of Al particles and encapsulate fluorinated graphene inside the coating layer. Thermal analysis results show that the coating layer can lower the reaction temperature of Al in the solid propellants due to the surface activation reaction between the Al and IL/FG. After substituting Al@IL/FG with Al, the residual Al content in the condensed combustion products of solid propellants decreased by 11.37%. In addition, compared with Al-based propellant, the d (0.5) of condensed combustion products of Al@IL/FG-based solid propellant was reduced from 69.157 to 21.559 μm. These results indicate that Al@IL/FG has a higher combustion efficiency than Al in solid propellants.

## 1. Introduction

Aluminum (Al) is widely used in solid propellants due to its high energy density and combustion heat value, which can enhance the combustion performance of the propellant [1,2,3,4]. However, due to the extremely high reactivity and easy oxidation property of Al, a dense layer of aluminum oxide (Al_2_O_3_) will rapidly form on the surface of Al. The Al_2_O_3_ layer possesses a high melting point (2054 °C) [5,6], which hinders the rapid progress of the combustion reaction [7]. The Al particles melt and coalesce to form large agglomerates leading to incomplete combustion, which reduces the combustion efficiency [8,9,10].

Techniques for tuning the combustion performances of solid propellants involve modifying the ratios of their different ingredients, adding high-energy-density additives, and using novel metal fuels [11,12,13,14]. Although these methods can somewhat improve the combustion performance of solid propellants, it is limited, due to the negative impact on safety or storage performances [15,16,17]. Alternatively, surface modification of existing metal fuels like Al is a widely used and effective method to improve the combustion performance [18,19,20,21]. For example, Al particles were used as the core and ammonium dinitramide (ADN) as the shell to prepare a core–shell structured micro-composite (Al@ADN) via in situ crystallization growth. It is found that the internal Al reacts with the acid produced by the decomposition of ADN, thereby corroding the oxide layer on the Al surface and ultimately enhancing the combustion performance of Al [22].

However, it is necessary to optimize the surface properties before modifying the Al particles, due to the lack of active functional groups on the Al surface. Rajarajeswari et al. employed an ionic liquid ([BMIM]BF_4_) to coat CuO nanoparticles, resulting in a marked reduction in particle size and a decrease in the bandgap, which facilitated an enhanced generation of reactive oxygen species [23]. Xu et al. developed a porous, superparamagnetic, and highly recyclable heterogeneous catalyst by applying a coating of poly(3-butylvinylimidazolium cobalt chloride) ionic liquid (P[BVIM]CoCl_3_) onto Fe_3_O_4_@SiO_2_ magnetic nanospheres [24]. Zhang et al. utilized symmetric imidazole ionic liquid coatings on Pd@ILs and Pt@ILs nanoparticles, functioning as quasi-homogeneous catalysts, which improved the thermal stability of fuels through the synergistic promotion of catalytic cracking and dehydrogenation reactions [25].

However, the above-mentioned ionic liquids modified with nanoparticles have lower energy, and using them as a coating layer for Al powder will reduce the theoretical specific impulse of the propellants. Imidazolium dicyanamide ionic liquids derivatives, recognized as a representative class of hypergolic ionic liquids (HILs), can spontaneously ignite upon contact with certain oxidizers, such as white fuming nitric acid [26,27]. HIL as a coating can be ignited first and then efficiently trigger the combustion of the inner aluminum core. In addition, using HIL coated Al powder can not only suppress the agglomeration of aluminum powder but also provide energy, which helps to improve the overall heat release and combustion efficiency [28]. In this study, we use ionic liquids (ILs) and fluorinated graphene (FG) to modify Al. IL polymerization can form a coating layer on the surface of Al powder, making it a suitable binder for aluminum powder. Moreover, the energy and gas products released by IL decomposition can promote the ignition of Al powder [29].

The use of fluorinated organic compounds (FCOs) in energetic materials is gaining growing interest [8,9]. Incorporating a small quantity of fluoropolymer into Al causes the fluoride to react with the oxide layer, which is conducive to etching away the Al_2_O_3_. Al particles without oxide layers have higher reactivity, allowing them to burn quickly in solid propellants, thereby improving the combustion efficiency [30]. Xiao et al. introduced a method of successfully preparing a dual core–shell structure by self-assembly of tannic acid (TA) and iron ions as a bridge. The Al@TA-Fe @PVDF composite material improves combustion and ignition performance. TA-Fe@PVDF, the interface layer, can ignite aluminum powder in oxygen at temperatures below its melting point [31]. Wang et al. constructed an ultra-thin and uniform single-layer fluorinated coating structure on the surface of aluminum powder through the self-assembly of fluorinated triblock copolymer G-F-G, utilizing the long-range attraction between fluorine fragments and aluminum [32]. FG is one of the commonly used FCOs, which can provide a higher liquidity of Al particles, enhancing the interfacial interaction [33]. Most importantly, fluorine atoms in FG are exposed on the Al surface and easily accessible for reacting with Al [34], thereby promoting combustion.

In this work, a core–shell structured Al-based composite (Al@IL/FG) was prepared. The structure and morphology of the Al@IL/FG was characterized. Then, the thermal decomposition results of Al@IL/FG with ammonium perchlorate (AP) were tested. The combustion performance of the Al@IL/FG composite was characterized as the solid propellant. Finally, the particle size of the condensed combustion products of Al@IL/FG-based solid propellant was used to characterize the combustion efficiency of Al powder.

## 2. Experimental Section

### 2.1. Materials and Instruments

Materials: Nano aluminum powder (average particle size 500 nm) was purchased from Evergrande Aluminum Industry Co., Ltd., Yongkang, Zhejiang Province, China, anhydrous ethanol (purity 99%) was purchased from Chengdu Jinshan Chemical Reagent Co., Ltd. Chengdu, Sichuan, China, vinyltrimethylsilane (VTMS) was purchased from Shanghai Bide Pharmaceutical Technology Co., Ltd., Shanghai, China, azodiisobutyronitrile (AIBN) was purchased from Chengdu Runze Local Chemical Co., Ltd. Chengdu, Sichuan, China, and fluorinated graphene was purchased from Shanghai Macklin Biochemical Co., Ltd., Shanghai, China. For the laboratory synthesis of ionic liquids (1-vinyl-3-butylimidazolium dicyanamide, [Amim][N(CN)_2_]), the specific synthesis steps are as follows [35].

Step 1: Synthesis of 1-vinyl-3-butylimidazolium chloride: 1-vinylimidazole (0.25 mmol, 23.56 g), bromon–butane (0.25 mmol, 34.18 g) and 200 mL acetonitrile were mixed in a 500 mL flask. After heating to 80 °C and full reflux reaction for 18 h, the reaction liquid was subjected to vacuum distillation to remove the excess reactants and solvent, and the crude product was obtained. These crude products were further separated and purified by column chromatography (dichloromethane/ethyl acetate: 1/2); then, the solvent was removed by vacuum distillation, further concentrated, and finally vacuum dried for 48 h.

Step 2: Synthesis of [Amim][N(CN)_2_]: First, 5.6 g Na[N(CN)_2_] and 10 g 1-vinyl-3-butylimidazolium chloride were added into 10 mL ethanol, and the suspension was stirred for 8 h in the dark. The suspension was filtered, and then, the yellow liquid was obtained by vacuum distillation. Finally, the product was dried in a vacuum with 92.2% yield.

Instruments: NEXSA X-ray Photoelectron Spectrometer (XPS), Thermo Fisher Scientific, Waltham, MA, USA. XRY-1A+ Series Oxygen Bomb Calorimeter, Shanghai Changji Geological Instrument Co., Ltd., Shanghai, China. X-ray Diffractometer (XRD), Rigaku Corporation, Tokyo, Japan. True Density Analyzer, Micromeritics Instrument Corporation, Norcross, GA, USA. ZEISS sigma5000 Scanning Electron Microscope (SEM), Carl Zeiss AG, Oberkochen, Germany. Synchronous Thermal Analyzer (TGA-DSC), Hengjiu Experimental Equipment Co., Ltd., Beijing, China. BRUKER XFlash 6130 Energy Dispersive Spectrometer (EDS), Bruker Corporation, Karlsruhe, Germany. Mastersizer 2000 Laser Particle Size Analyzer, Malvern Instruments, Malvern, UK. IX high-speed camera, Beijing Juncheng Technology Co., Ltd., Beijing, China.

### 2.2. Preparation of Al@IL/FG

**Figure 1:**
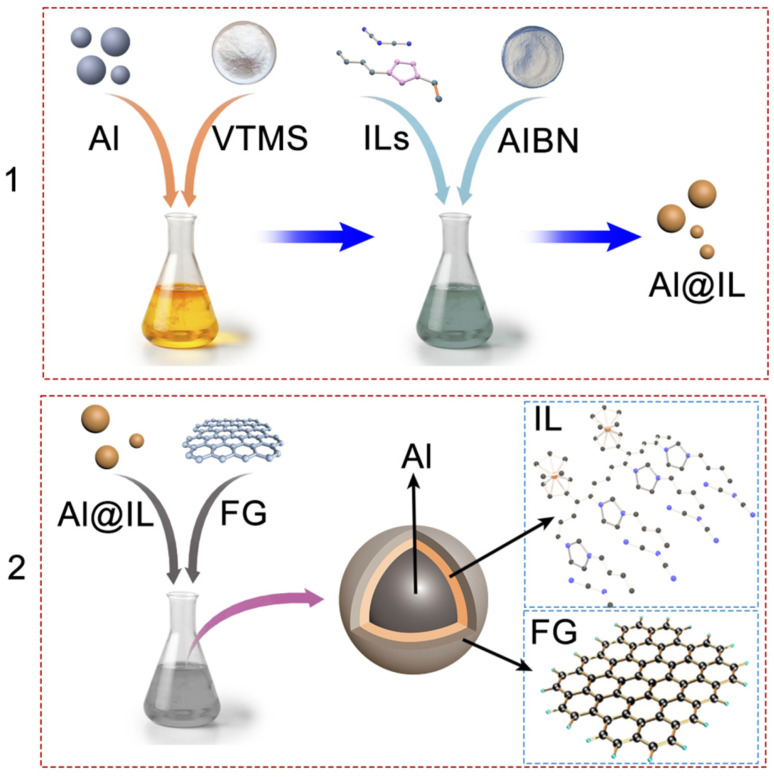
Al@IL/FG preparation process diagram.

The experimental procedure is illustrated in Figure 1. Nano-aluminum (5 g), VTMS (0.5 g) and 50 mL of ethanol were added into the 100 mL beaker and stirred for 4 h. Then, 0.5 g [Amim][N(CN)_2_] and 0.01 g AIBN were added into the mixture and stirred for 2 h at 70 °C. After that, 0.03 g FG was added, and the mixture was stirred at 70 °C for 5 h. Finally, the Al@IL/FG composite was obtained by filtering and vacuum drying.

### 2.3. Preparation of Propellants

The mixture components of Al@IL/FG composites, AP, binder and others were added into the stirring cup in certain proportions (as shown in Table 1) and stirred as the uniform gelatinous slurry. Then, the propellant slurry was injected into the mold. The slurry was cured at 65 °C for 7 d. The preparation of Al-based solid propellants followed the same method.

### 2.4. Characterization of the Al@IL/FG

The surface structure and morphology of Al@IL/FG and Al@IL particles were characterized using XPS, XRD, and SEM. The elemental content and distribution of the particles were verified using EDS. TG-DSC was employed to evaluate the thermal decomposition properties of the composites, heating from 40 °C to 1000 °C at a rate of 10 °C/min under an argon flow rate of 60 mL/min. The burning rate of the samples was calculated using a high-speed camera with 2000 fps.

### 2.5. Characterization of Composite Solid Propellants

The thermal decomposition properties of the solid propellants were tested by TG-DSC at the heating rate of 10 °C/min. The combustion performances were tested by high-speed camera (i-SPEED-7) with 1000 fps. A high-speed infrared thermal imager (3841640H) was used to test the combustion temperature of propellants with 1000 fps. The condensed combustion products of solid propellants were collected and analyzed by SEM, XRD, and a laser particle size analyzer.

## 3. Results and Discussion

### 3.1. Structural Properties of Al@IL and Al@IL/FG

**Figure 2:**
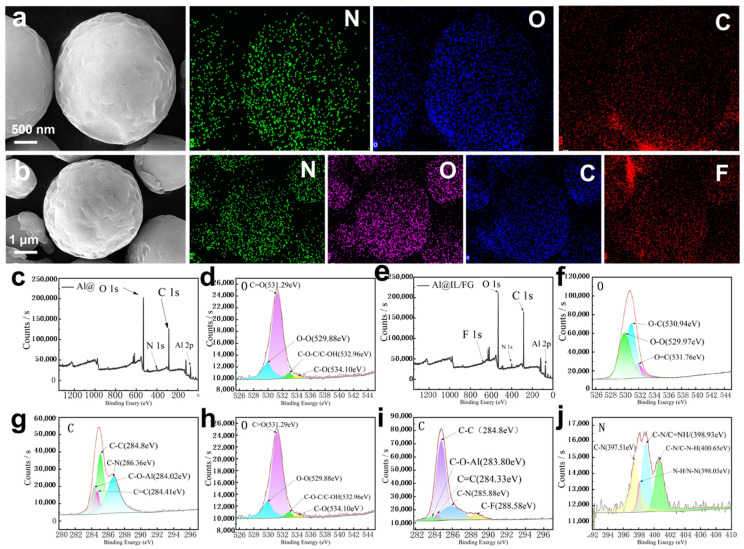
SEM and EDS images of Al@IL (**a**) and Al@IL/FG (**b**) and XPS curves of Al@IL (**c**–**f**) and Al@IL/FG (**g**–**j**).

The morphology of Al@IL and Al@IL/FG was examined by SEM. Ionic liquids (IL) are used as a binder, which enable the FG to adhere onto the surface of Al particles. As shown in Figure 2a,b, both the Al@IL and Al@IL/FG surfaces are covered in wrinkled film. This is the result of IL acting as the binder that connects Al and FG. EDS analysis demonstrated that C is evenly distributed in Al@IL and Al@IL/FG, indicating that the IL is successfully coated onto the surface of the Al particles. The distribution of F indicates that FG is combined with Al@IL and has formed Al@IL/FG.

XPS was used to further investigate the elemental state, as shown in Figure 2c,d. Both Al@IL and Al@IL/FG exhibited distinct signal peaks in their XPS spectra. Specifically, three characteristic peaks at 284.0, 400.1 and 531.4 eV were identified, corresponding to C1s, N1s and O1s, respectively. Al@IL/FG displays unique F1s peaks, indicating the existence of FG. These results indicate the successful coated of FG on Al particles.

As shown in Figure S1, the XRD spectral peak data of Al, Al@IL, and Al@IL/FG are identical. This suggests that the coatings on the Al surface do not alter the morphology of the Al particles. Additionally, because the amounts of IL and FG coating layers on the aluminum surface are relatively small, their characteristic peaks are not clearly visible in the XRD results.

### 3.2. Thermal Properties

**Figure 3:**
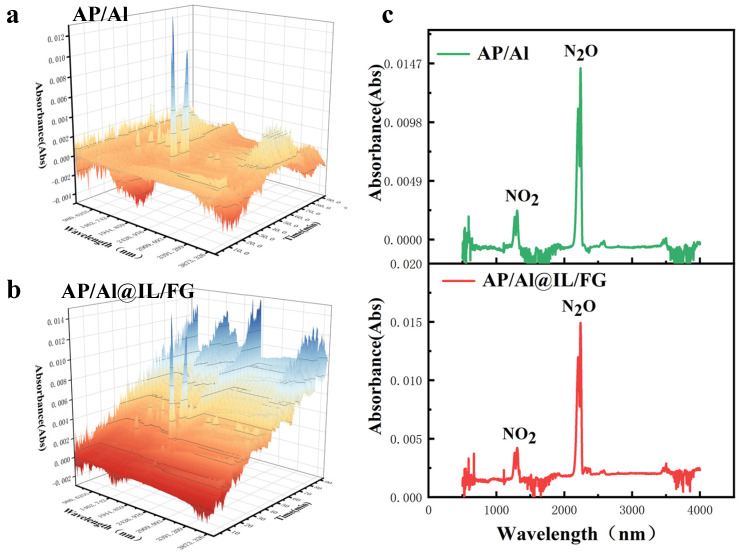
FTIR spectra of AP/Al (**a**) and AP/Al@IL/FG mixtures (**b**); infrared absorption peak of thermally decomposed nitrogen oxides (**c**).

**Figure 4:**
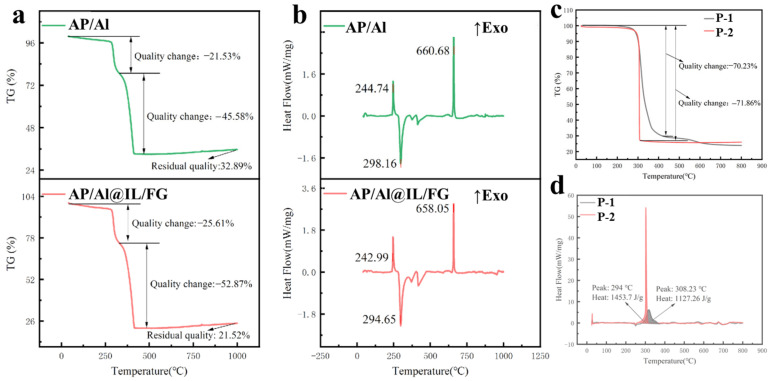
The TG (**a**) and DSC (**b**) curves for Al and Al@IL/FG and the TG (**c**) and DSC (**d**) curves for their corresponding propellants.

In order to understand the influence of IL and FG on the thermal reaction of Al powder, the gaseous products during the thermal decomposition process were further analyzed. The nonisothermal thermal decomposition process was monitored by FTIR online with a heating rate of 10 K/min. Figure 3 shows the infrared spectrum of the thermal decomposition process, and Figure 3c represents the FTIR spectra of gas products produced from AP/Al and the AP/Al@IL/FG mixture at the time when the intensity of FTIR series was the maximum. According to the relevant literature reports [36], it was certain that there were N_2_O (2238 cm^−1^ and 2201 cm^−1^), NO_2_ (1630 cm^−1^ and 1598 cm^−1^), H_2_O (3500–4000 cm^−1^), NO (1908 cm^−1^), HCl (2700–3012 cm^−1^), HNO_3_ (1710 cm^−1^) and HClO_4_ (1120 cm^−1^) for both compositions. The main gas products were N_2_O and NO_2_ based on the absorbance intensity. As shown in Figure 4c, the absorption intensity of N_2_O was higher than that of NO_2_, indicating that the reaction of N_2_O was dominant in the thermal decomposition process at that time. The absorption intensity of N_2_O and NO_2_ produced by AP/Al was lower than that of AP/Al@IL/FG, which indicates that the latter produces more of the two gases and has less residual mass than the former.

The thermal reactivity of the Al@IL/FG was studied by TG-DSC. All the samples were mixed with 20%wt AP. As shown in Figure 3a, when Al@IL/FG and Al were mixed with AP, respectively, there were two stages of mass loss that corresponded to the low-temperature (250~390 °C) decomposition of AP, which showed that the mass loss during the low-temperature decomposition stage was caused by heterogeneous solid gas reactions [10]. The first and second stages of mass loss occurred at 250~300 °C and 300~380 °C, respectively, which corresponded to the low-temperature decomposition of AP. Further analysis was conducted on the proportion of quality loss in the two decomposition stages. Al exhibited two stages of mass loss at 21.53% and 45.58%, with a residual mass of 32.89%; Al@IL/FG showed two stages of mass loss at 25.61% and 52.87%, with a residual mass of 21.52%. Less residue indicates that the aluminum powder particles modified by ionic liquid and fluorinated graphene are decomposed more completely. During the thermal decomposition process, more gaseous products were produced, resulting in an increase in quality loss, a significant decrease in residual mass, and a significant decrease in agglomeration trend. In Figure 4b, the exothermic peak temperature of Al was 298.16 °C, while the exothermic peak temperature of Al@IL/FG was 294.65 °C. The latter shows a slightly lower decomposition temperature. This indicates that the modified aluminum powder has a thermal catalytic effect on the thermal decomposition process of AP.

Figure 4c,d show the TG and DSC curves for P-1 and P-2, respectively. The thermal decomposition curve parameters of the two propellants are listed in Table 2. The exothermic peak temperature of P-1 is 308.23 °C, releasing 1127.26 J·g^−1^ heat. The corresponding TG stage loss weight is 70.23%. The exothermic peak temperature of P-2 is 294 °C, the heat absorption is 1453.70 J·g^−1^, and the mass loss is 71.86%. Compared with the weight loss stage of TTJ^−1^, the weight loss process time was reduced, the exothermic peak was advanced, and the heat absorption was higher.

**Figure 5:**
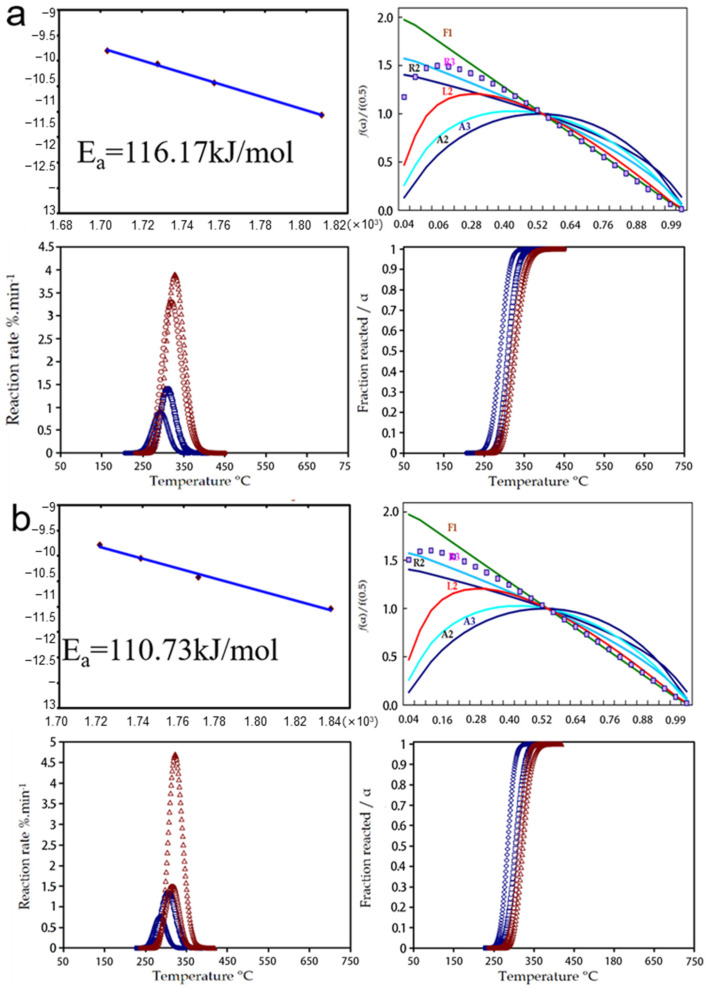
Reaction kinetics and kinetic model diagrams of AP/Al (**a**) and AP/Al@IL/FG (**b**).

In chemical reactions, the reaction with a smaller activation energy is easier to obtain [37]. Therefore, the activation energy of AP/Al@IL/FG and AP/Al was tested with different heating rates. The results are shown in Figure 5. The fitting equation for Al obtained from the fitting results in the figure is Y = −12,285X + 10.648, with a correlation coefficient of R^2^ = 0.9989. The calculated thermal decomposition activation energy E_a_ = 116.17 kJ/mol. The fitting equation for Al@IL/FG is Y = −11,184X + 8.9594, with a correlation coefficient of R^2^ = 0.9925. The calculated activation energy for thermal decomposition is E_a_ = 110.73 kJ/mol. The activation energy of AP/Al@IL/FG is lower than that of Al, and its reaction rate is faster. The kinetic models of AP/Al and Al@IL/FG are closer to the F1 model.

### 3.3. Combustion Characteristic

**Figure 6:**
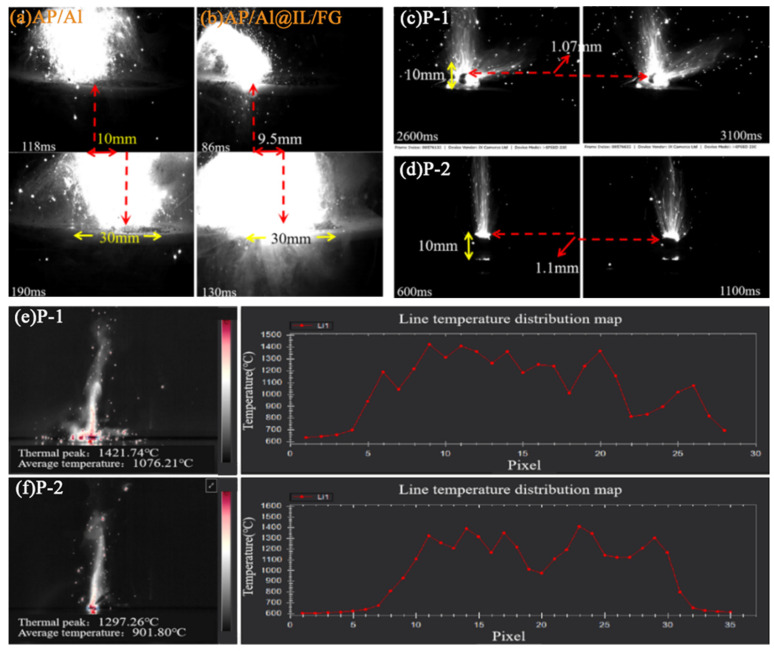
Combustion image of AP/Al (**a**), AP/Al@IL/FG (**b**), P-1 (**c**) and P-2 (**d**) during the burning speed test process. (**e**,**f**) Combustion temperatures of P-1 and P-2.

The burning rate is an important indicator for evaluating the combustion performance. To investigate the differences in burning rate between Al and Al-based composite materials, Al and Al@IL/FG were mixed uniformly at a 1:1 mass ratio with AP. The total test lengths of Al and Al@IL/FG were 33 mm and 20 mm, respectively. The burning rate tests used a high-speed camera with 2000 fps. As shown in Figure 6, the burning rate of Al is 152.78 mm/s, and the burning rate of Al@IL/FG is 158.5 mm/s. The results indicate that the low difference in the burning rate of Al and Al@IL/FG is due to a little IL and FG on the surface of Al.

To study the influence of Al@IL/FG on the combustion process of solid propellants, the burning rate and combustion temperature of solid propellants is shown in Table 3. Among them, Al is used as the metal fuel for P-1 and Al@IL/FG for P-2. The burning rate tests of P-1 and P-2 were conducted using a high-speed camera with 1000 fps. The burning rate of P-1 is 2.14 mm/s. The combustion process of P-1 is accompanied by a large number of visible particle aggregates. This is due to the significant agglomeration of Al-based propellants during combustion. Similarly, the burning rate of P-2 is 2.19 mm/s, which is close to that of P-1. But during the combustion, the agglomeration of Al weakened in P-2, and the size of the aggregates decreased. This is because a certain degree of reaction occurred between IL/FG and the oxide layer on the surface of Al, effectively avoiding the formation of Al_2_O_3_ on the surface of Al powder. This reaction effectively prevented the formation of Al_2_O_3_ on the surface of Al powder. The infrared temperature distribution maps and line temperature distribution maps, shown in Figure 6e,f, are analyzed, respectively. The line temperature distribution map of P-2 was closer to a normal distribution, and the combustion temperature fluctuation of P-1 was higher. This result indicates that the combustion of P-2 is more stable.

### 3.4. Condensed Combustion Products Analyses

**Figure 7:**
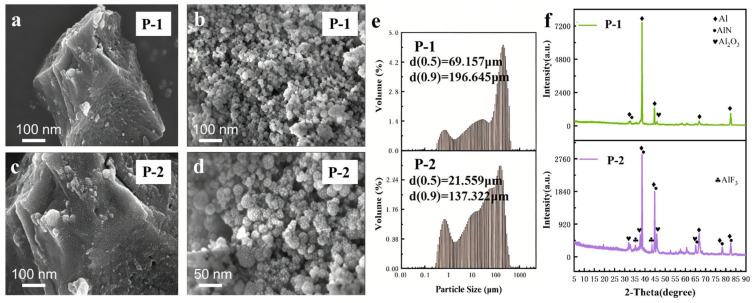
SEM images of condensed combustion products of (**a**) and (**b**) P-1 and (**c**) and (**d**) P-2, (**e**) particle size diagram and (**f**) XRD pattern of condensed combustion products of P-1 and P-2.

In order to further analyze the combustion efficiency of metal fuels, the condensed combustion products of propellants are characterized. Figure S2 and Figure 7 present the SEM images of the condensed combustion products of P-1 (Figure 7a,b) and P-2 (Figure 7c,d), respectively. It can be seen that the condensation combustion products of P-1 contain bulk sintered aggregates with a size of about 1 μm, which are relatively large. Not only is the Al not completely burned, but the increase is a two-phase flow loss. The result reduces the combustion efficiency of Al and decreases the engine performance. The condensed combustion products of P-2 contain sintered aggregates of approximately 50 nm in size, and these can be identified as spherical particles. Compared with P-1, the size of the condensed products is reduced. This result indicates that the coating layer can effectively reduce the aggregation of Al.

In order to further verify the effect of the coating layer on the particle size of condensation combustion products, the laser particle size distribution measurement method is applied to study the particle size distribution of condensation combustion products of the propellant. As shown in Figure 7e, d (0.5) is the average radius of the condensation combustion products, and d (0.9) is the diameter of large particles. The d (0.5) and d (0.9) of the condensation combustion products of P-1 were 69.157 μm and 196.645 μm, respectively. The d (0.5) and d (0.9) of the condensation and combustion products of P-2 were 21.559 μm and 137.322 μm, respectively. There were significant changes in d (0.5) and d (0.9) for P-2 compared to P-1, where d (0.5) was reduced by about 3.2 times, and d (0.9) was reduced by about 1.4 times. This result indicates that the large aggregates produced by P-2 combustion were reduced, and the average size of the aggregates was also reduced.

Then, XRD was used to investigate the compounds present in the condensation combustion products, as shown in Figure 7f. The results show that the concentrated combustion products of P-1 are mainly composed of Al, Al_2_O_3_ and Al(NO_3_)_3_, and the content of Al and Al_2_O_3_ is relatively high. The concentrated combustion products of P-2 mainly include Al, Al_2_O_3_, Al(NO_3_)_3_ and AlF_3_. Compared with P-1, the content of Al(NO_3_)_3_ in the concentrated combustion product of P-2 is increased. This indicates that the conversion of Al to Al(NO_3_)_3_ was improved. In addition, the result implies that FG can catalyze Al, forming Al(NO_3_)_3_ and AlF_3_. More Al is involved in the reaction, which reduces the generation of aggregates and improves the combustion efficiency of the propellant.

## 4. Conclusions

This study explores the influence of Al-based composites on the combustion performance of solid propellants. The results indicate that IL enables FG to be uniformly coated on the surface of Al particles and form wrinkles, without altering the crystal structure of aluminum during the entire coating process. In the two stages of thermal decomposition, there was a significant difference in the mass loss and residual mass of Al@IL/FG compared to Al, with both showing an increase in mass loss. However, the residual mass of Al@IL/FG decreased by 11.37% compared to Al. This indicates that the combustion of Al@IL/FG is more thorough, and the agglomeration is reduced. Then, the burning rate of P-2 based on Al@IL/FG was basically the same as that of P-1, due to the very small amounts of IL and FG. In addition, the thermal decomposition peak of P-2 was slightly earlier than P-1, and the heat release increased by 326.44 J·g^−1^, proving that Al@IL/FG can lower the reaction temperature of propellants. Al@IL/FG can reduce the size of agglomerates produced by the combustion of propellants. The diameter of condensed combustion products of P-2 is reduced by about 1.4 times that of P-1, while the average radius of the condensed combustion products of P-2 is reduced by about 3.2 times that of P-1. This indicates that the modified aluminum powder can inhibit the formation of agglomerated particles during combustion and reduce the risk of clogging solid rocket engines.

## Figures and Tables

**Table 1:** The propellant formulations.

Sample	Al	Al@IL/FG	AP	BUNENA	IPDI	HTPB
P-1	18%	-	64%	0.5%	0.5%	17%
P-2	-	18%	64%	0.5%	0.5%	17%

**Table 2:** DSC parameters of different samples and propellants.

Sample	Exothermic Peak Temperature (°C)	Heat Release (J·g^−1^)	Mass Loss (%)	Residual Mass (%)
AP/Al	298.16	-	67.11	32.89
AP/Al@IL/FG	294.60	-	78.48	21.52
P-1	308.23	1127.26	70.23	29.77
P-2	294.00	1453.70	71.86	28.14

**Table 3:** Burning rate and combustion temperature of propellant (The average of all data was taken after being tested five times).

Sample	Burning Rate (mm/s)	Burning Temperature (°C)
P-1	2.14 ± 0.20	1430 ± 68
P-2	2.19 + 0.15	1280 ± 43

## Data Availability

All the data and information relevant to the article are present within the article itself.

## Supplementary Materials

The following supporting information can be downloaded at https://www.mdpi.com/article/10.3390/nano16120757/s1, Figure S1: XRD spectra of Al, Al@IL, and Al@IL/FG; Figure S2: Images of different propellants.
