# Supplementary material for: Improving the Combustion Efficiency of Aluminum-Based Composite, Al@IL/FG, Through Surface Activation Reaction
Source: Nanomaterials (Basel). 2026 Jun 16;16(12):757. doi: 10.3390/nano16120757 (PMC13305099; doi:10.3390/nano16120757)
Supplement: Supplementary file 1 [file nanomaterials-16-00757-s001.zip › nanomaterials-4327102-supplementary.pdf]

Improving the Combustion Efficiency of Aluminum-Based Composites Al@IL/FG Through Surface Activation Reaction

Qi-long Zheng [1,*], Zhi-lei Huang [2], Hui-xiang Xu [1], Ji-Zhen Li [1] and Wei He [2,*]

1 Xi'an Modern Chemistry Research Institute, Xi'an 710065, China; xhx204@163.com (H.-x.X.); jizhenli@126.com (J.-Z.L.)

2 Key Laboratory of Advanced Spatial Mechanism and Intelligent Spacecraft, Ministry of Education, School of Aeronautics and Astronautics, Sichuan University, Chengdu 610065, China

* Correspondence: zhenglong2577@163.com (Q.-l.Z.); hewei_91@scu.edu.cn (W.H.)

Table of Contents

1.XRD spectra of Al, Al@IL, and Al@IL/FG 2

2.SEM images of different propellants 3

1.XRD spectra of Al, Al@IL, and Al@IL/FG

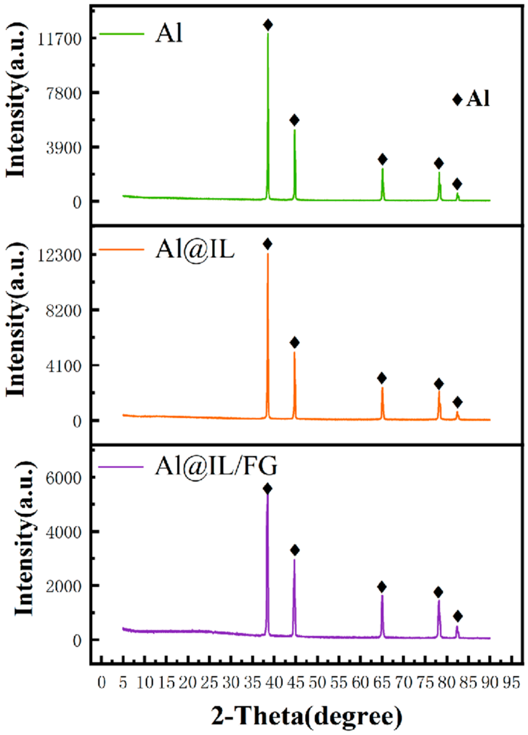

Figure S1: XRD spectra of Al, Al@IL, and Al@IL/FG.

2.SEM images of different propellants

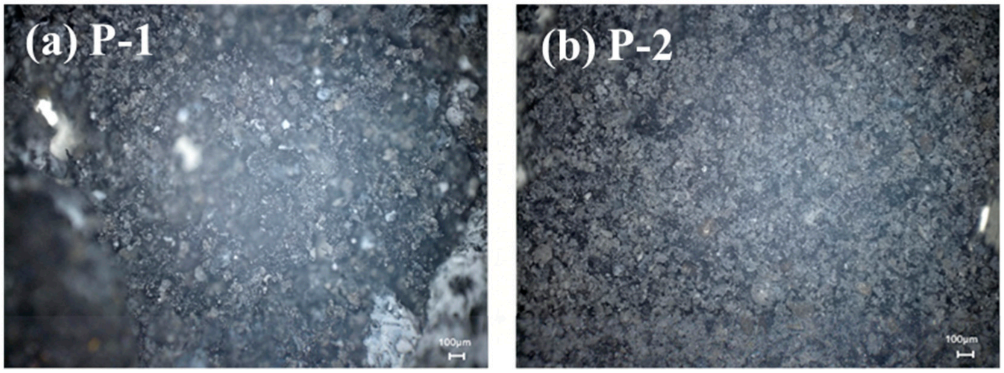

Figure S2: Images of different propellants.
